# Ontario Veterinary Association, Toronto, Canada

**Published:** 1896-02

**Authors:** C. H. Sweetapple

**Affiliations:** Secretary


### ONTARIO VETERINARY ASSOCIATION, TORONTO, CANADA.

THE annual meeting of this Association was held in the Veterinary College, Toronto, on Friday, December 21, 1895.

Mr. G. L. Robson, V.S., the President, opened the meeting with a most excellent address, containing many points of practical interest and much food for thought.

After the reading of the minutes of the last meeting, the Secretary's and Registrar's reports, and communications, a discussion ensued relating to the New Act respecting Veterinary Surgeons, passed in 1895, and a fine having been imposed under that Act upon a person for falsely claiming the title, which fine should have been paid over to the funds of this Association.

The following new members were duly elected: W. J. Morgan, V.S.; F. G. Hutton, V.S.; and J. Wagner, V.S.

Prof. Sisson, of the Ontario Veterinary College, read an excellent paper on "Undescended Testicle in the Horse." In the discussion that followed, Mr. Quinn, V.S., who has had much experience in the castration of "Cryptorchids," warmly complimented Mr. Sisson on the excellence of his anatomical description of the condition existing.

Mr. A. Crowforth read a very interesting paper on "Toxins and Antitoxins in Relation to the Cause, Cure, Prevention, and Diagnosis of Disease."

It was resolved that the Secretary should be instructed to get new copies of the Register printed, and to have incorporated in the pamphlet the "Act respecting Veterinary Surgeons," passed in 1895. These to be printed after April 1st of the incoming year, and a copy to be forwarded to each member of the Association.

The following are the officers for the ensuing year: Honorary President, Prof. A. Smith, F.R.C., V.S.; President, H. Hopkins, V.S.; First Vice-President, Major Lloyd; Second Vice-President, S. Sisson, V.S.; Secretary, C. H. Sweetapple, V.S.; Treasurer, W. Cowan, V.S.; Directors, J. Wende, W. J. Wilson, W. Morgan, J. F. Quinn, W. Steele, W. Gibb, A. Crowforth, and W. Burns; Auditors, C. Elliott and J. D. O'Neil; Delegates to the Western Fair Association, J. H. Wilson and J. D. O'Neil; Delegates to the Industrial Exhibition Association, Toronto, Prof. A. Smith and H. Hopkins. Mr. W. Cowan was nominated to represent the Association at the Central Farmers' Institute, but he explained that it would be better for the veterinary surgeons practising in the various localities to be placed on the lecturing staff.

C. H. SWEETAPPLE,
Secretary.

